# P-1976. Comparing Clinical Expertise and Chat-GPT in the Management of Septic Shock and Severe Pneumonia: A Pilot Study

**DOI:** 10.1093/ofid/ofaf695.2143

**Published:** 2026-01-11

**Authors:** Rhea Bohra, Jassimran Singh, Eric Silverman, George M Abraham, Sophee Niraula, Aditi Luitel, Smriti Dhakal, Sushaili Pradhan, Rajaeswaran Chinnamuthu

**Affiliations:** Saint Vincent Hospital, Worcester, MA; Saint Vincent Hospital, Worcester, MA; Saint Vincent Hospital, Worcester, MA; Saint Vincent Hospital, Worcester, MA; Memorial Sloan Kettering Cancer Center, MANHATTAN, New York; Albert Einstein College of Medicine, BRONX, New York; Chicago Public Schools, CHICAGO, Illinois; Care New England Health System, PAWTUCKET, Rhode Island; Saint Vincent Hospital, Worcester, MA

## Abstract

**Background:**

The evolution of artificial intelligence (AI) and large language models (LLMs) offers promising opportunities in infection management. Sepsis identification using AI has been integrated into many Electronic Medical Recording systems and applications for diagnostics and antimicrobial stewardship are emerging. This pilot study assessed ChatGPT-4® as a clinical decision aid for the management of septic shock and severe pneumonia.

**Figure:**
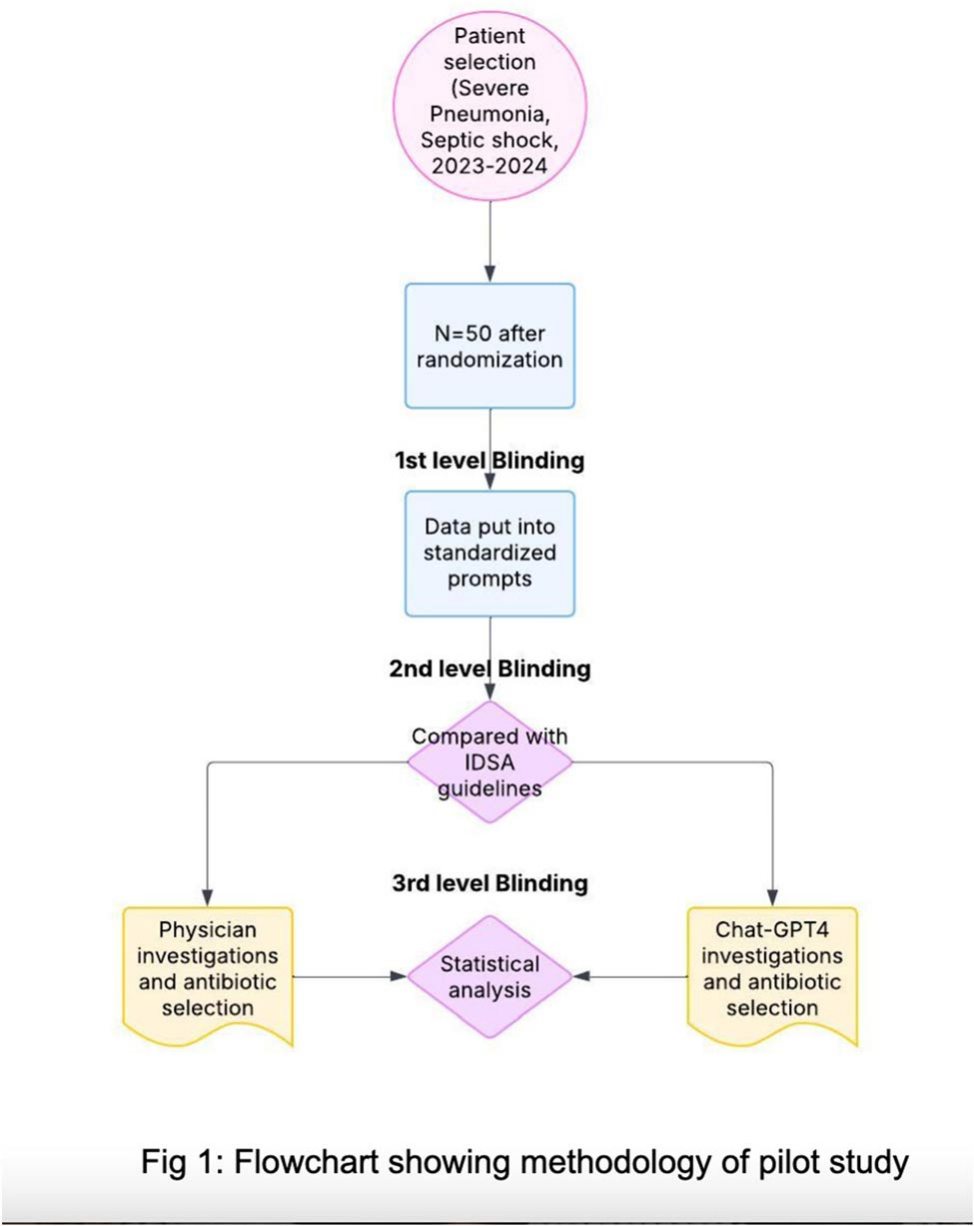
Flowchart showing methodology of pilot study

**Figure:**
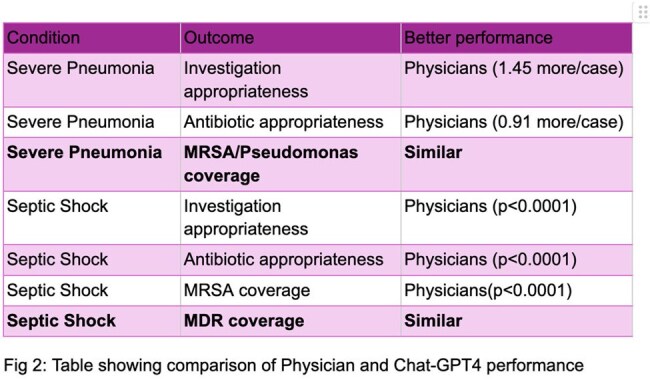
Table showing comparison of Physician and Chat-GPT4 performance

**Methods:**

A retrospective study was conducted at Saint Vincent Hospital, Worcester on 50 cases (2023-2024). Physician-documented investigations and antibiotics were compared with ChatGPT-4® outputs generated using standard prompts with full blinding. Infectious Diseases Society of America guidelines served as the standard. Paired t-tests and McNemar’s test evaluated investigation appropriateness and antibiotic selection accuracy respectively, using Statistical Analysis Software® Version 9.4.

**Fig 3:**
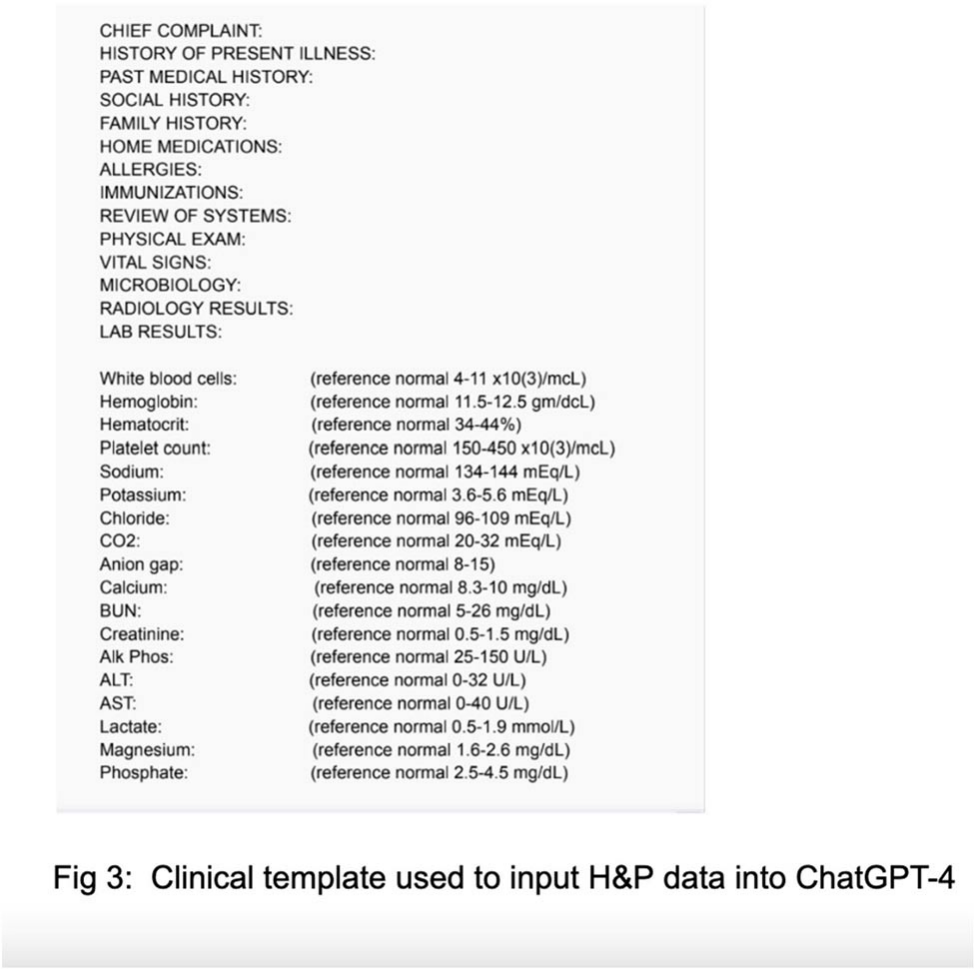
Clinical template used to input H&P data into ChatGPT-4

**Results:**

Severe Pneumonia: Physicians recommended more appropriate investigations (mean difference 1.45 out of 4 tests) and antibiotics (0.91 more/case) compared to ChatGPT-4®, with no significant difference in *MRSA* or *Pseudomonas* coverage.

Septic Shock: Physicians outperformed ChatGPT-4® in investigations (mean difference 1 out of 3 tests) and antibiotic choices (1 more/case), with greater accuracy for *MRSA* coverage; concordance was noted for Multi-Drug Resistant (MDR) organisms.

**Conclusion:**

Physicians outperformed ChatGPT-4 across both conditions. However, ChatGPT-4® demonstrated comparable pathogen-specific antimicrobial selection and accuracy in MDR coverage, suggesting potential with diagnosis-specific prompting and antimicrobial stewardship.

The results highlight the enduring importance of physician-led decision-making in an era increasingly shaped by AI. LLMs still face limitations including data privacy concerns and need for individualized contextual judgement that prevent their autonomous use.

However, with refinement and responsible implementation, LLMs may evolve into a trusted aid to enhance physician decision-making, especially in areas with limited access to specialist care.

This study has led to a prospective trial exploring ChatGPT-4’s use with real-time, targeted prompts throughout hospitalization.

**Disclosures:**

All Authors: No reported disclosures

